# B Cells and Autoantibodies in Multiple Sclerosis

**DOI:** 10.3390/ijms160716576

**Published:** 2015-07-21

**Authors:** Anne-Katrin Pröbstel, Nicholas S. R. Sanderson, Tobias Derfuss

**Affiliations:** 1 Department of Neurology, University Hospital Basel, Petersgraben 4 & Clinical Neuroimmunology, Hebelstrasse 20, 4031 Basel, Switzerland; E-Mails: anne-katrin.proebstel@usb.ch (A.-K.P.); nicholas.sanderson@unibas.ch (N.S.R.S.); 2 Department of Biomedicine, University of Basel, Hebelstrasse 20, 4031 Basel, Switzerland

**Keywords:** multiple sclerosis, neuromyelitis optica, B cells, autoantibodies, autoantigen, pathogenesis, therapy

## Abstract

While over the past decades T cells have been considered key players in the pathogenesis of multiple sclerosis (MS), it has only recently become evident that B cells have a major contributing role. Our understanding of the role of B cells has evolved substantially following the clinical success of B cell-targeting therapies and increasing experimental evidence for significant B cell involvement. Rather than mere antibody-producing cells, it is becoming clear that they are team players with the capacity to prime and regulate T cells, and function both as pro- and anti-inflammatory mediators. However, despite tremendous efforts, the target antigen(s) of B cells in MS have yet to be identified. The first part of this review summarizes the clinical evidence and results from animal studies pointing to the relevance of B cells in the pathogenesis of MS. The second part gives an overview of the currently known potential autoantigen targets. The third part recapitulates and critically appraises the currently available B cell-directed therapies.

## 1. Introduction

Multiple sclerosis (MS) is a chronic inflammatory disease of the central nervous system (CNS). Both experimental and clinical evidence suggest that it is initiated by autoreactive immune cells directed against components of the CNS, be it the oligodendrocytes, the astrocytes, or the neurons [1]. The pathologic hallmarks are demyelination, gliosis, and axonal loss, the latter of which is thought to contribute most to sustained disability [2]. Despite numerous experimental, genetic, and epidemiological studies, the trigger mechanisms of this autoimmune disorder remain elusive. MS is thought to be caused by a complex interplay of genetic and environmental factors (infections, Vitamin D, gut microbiome, and others) [1,3,4,5,6]. One of the potential and most controversially discussed infectious triggers is Epstein-Barr virus infection, which might lead to cross-reactive antibodies targeting CNS autoantigens [7,8]. Having been considered for a long time as a T cell-dominated disease, based on the T cell-driven animal model of experimental autoimmune encephalomyelitis (EAE), B cells have moved into focus over the recent years, inspired by the success of B cell-directed therapies [1,9,10] and emerging experimental evidence of direct B cell involvement extending far beyond their role as mere antibody-producing cells [2,11]. This review summarizes the clinical evidence and results from animal studies pointing to the relevance of B cells in the pathogenesis of MS. It also gives a detailed overview of the currently known potential autoantigen targets. This knowledge provides the basis to understand the rationale behind B cell-directed therapies that are discussed in the third part.

## 2. The Many Faces of B Cells in MS—Beyond Antibody Production

### 2.1. Clinical Evidence for B Cell Involvement in MS

Various findings in patients with MS suggest the involvement of B cells in the pathogenesis, including: the presence of oligoclonal bands (OCBs), clonal expansion of B cells in the cerebrospinal fluid (CSF), antigen-dependent affinity maturation of antibodies, immunoglobulin (Ig) and complement deposition in lesions, the presence of B cell follicle-like structures, and a B cell-fostering milieu.

#### 2.1.1. Oligoclonal Bands (OCBs), Clonal Expansion, and Antigen-Driven Affinity Maturation of B Cells

OCBs are one of the few biomarkers used in clinical practice to establish the diagnosis of MS [1,3,4,5,6,12]. OCBs are clonally expanded antibodies that are produced intrathecally and are not found in serum [13]. The presence of OCBs is very stable over time, albeit with substantial modulation by a few immunomodulatory treatments [14,15]. To date, several attempts have failed to identify the target antigens of the OCBs [16]. Comparison of the OCB proteome with the transcriptome of B cells in the CSF revealed that rearranged Ig sequences in most B cells in the CSF are represented by peptides found in the OCBs, allowing the conclusion that, indeed, clonally expanded B cells in the CSF contribute to the production of the OCBs [17]. Moreover, the transcriptome of B cells in the CSF overlaps with those in MS lesions, indicating a functional relationship between B cells in the CSF and brain parenchyma [18]. Even more interestingly, molecular analysis of B cells in the CSF and MS lesions revealed not only clonal expansion, but also somatic hypermutation, pointing toward antigen-driven stimulation [19,20,21]. Several recent studies have shown that in addition to intrathecal clonal expansion and somatic hypermutation of B cells in the CSF, there is trafficking of clonally related B cells between the peripheral and CNS compartments with antigen-driven maturation both in the periphery (cervical lymph nodes) and CNS [22,23,24,25]. All in all, there is increasing evidence for an active axis between the B cells found in the peripheral blood, lymph nodes, CSF, and MS lesions.

#### 2.1.2. Ig and Complement Deposition in Lesions and B Cell Follicle-Like Structures

The presence of Ig and complement deposition in at least a subtype of acute demyelinating MS lesions (Type II lesion) is a well-recognized phenomenon [26,27], and more recently, B cell follicle-like structures have been described in the meninges of patients with (mainly progressive) MS [28,29,30,31]. Furthermore, it has been shown in patients with progressive MS that these extra parenchymal meningeal B cell clones are related to those in the parenchyma and that the immunoglobulin G (IgG) repertoires of the brain lesion also connect to those in the CSF [18,32]. While until now the brain was considered as an “immune privileged organ”, it has only been shown very recently that lymphatic vessels are indeed present in the CNS and drain directly into cervical lymph nodes where CSF-derived dendritic cells are mainly clustered in B cell follicles [33,34]. Future research will have to show how MS lesions and B cell follicle-like structures relate to these newly discovered lymphatic vessels.

#### 2.1.3. B Cell Fostering Milieu: The BAFF/APRIL System

There is ample evidence of the presence and clonal relationship of B cells in the brain parenchyma, the meninges, and the CSF, and the chronic persistence of these cells presupposes a B cell fostering milieu. The B cell survival factor tumor necrosis factor (TNF) superfamily 13b (BAFF), the B cell-attracting chemokine (CXCL13), and the Chemokine (C-C motif) ligand 19 (CCL19) have been identified in the CSF and lesions of MS patients and are proposed to be key attractants [35,36,37]. In a relapsing experimental autoimmune encephalomyelitis (EAE ) model, intracerebral expression of BAFF and CXCL13 led to the formation of lymphoid follicle-like structures in the meninges [38]. Increased CXCL13 and CCL19 levels were also linked to intrathecal immunoglobulin production as well as the presence of B cells, plasmablasts, and T cells in MS patients [36,37]. Interestingly, expression of CXCL13 in CSF of MS patients was correlated with future inflammatory disease activity, thereby indirectly linking B cell recruitment and MS disease activity [39].

### 2.2. Experimental Evidence for B Cell Involvement in MS

A growing body of evidence for B cell involvement in MS from both clinical and human tissue-based studies, and more recently, animal models, is starting to shed light on the diverse functions of B cells, particularly the capacity of B cells to prime and regulate T cell responses in MS.

#### 2.2.1. B Cells as Antigen-Presenting Cells and Pro-Inflammatory Mediators

While the experimental model for MS, *i.e.*, the adoptive transfer of activated encephalitogenic T cells leading to demyelination, promoted the notion that MS is a T cell-mediated disease for a long time, it was recently elegantly shown that the B cell antigen-presenting (APC) function is crucial for the induction of myelin oligodendrocyte glycoprotein (MOG)-induced EAE. This was done by creating mice with a selective deficiency of major histocompatibility complex (MHC)II on B cells (B-MHC II(-/-)) [40]. B cells of these mice could still produce autoantibodies but were not able to stimulate T cells anymore. This reduced stimulatory capacity led to resistance to EAE induction. Moreover, it was shown that CNS-resident B cells can fuel inflammation, which is mediated by the release of pro-inflammatory cytokines namely TNF, lymphotoxin and interleukin-6 (IL-6), by re-activating infiltrating T cells. This effect is partly alleviated by B cell-depleting therapy [41,42,43].

#### 2.2.2. Regulatory and Anti-Inflammatory Capacities of B Cells

The involvement of B cells gets even more complex when one considers their capacity to down-regulate immune responses. This regulatory function involves both cell-mediated mechanisms and the secretion of anti-inflammatory cytokines such as IL-10 and IL-35 [43,44,45,46]. In mice, regulatory B cells have been phenotyped as immunoglobulin M (IgM)-positive, CD138-high, TNF receptor superfamily 13b-positive, CXC receptor-4-positive, CD1d-intermediate, and hepatitis A virus cellular receptor (T-cell mucin 1)-intermediate (IgM^+^, CD138^hi^, TACI^+^, CXCR4^+^, CD1d^int^, and TIM1^int^) [45]. Moreover, the generation of regulatory B cells in mice has been shown to depend in particular on the presence of IL-21 and T cell interaction via CD40/CD40L [47].

## 3. Autoantigen Candidates in MS—The Ongoing Treasure Hunt

### 3.1. In Vitro and in Vivo Evidence for Autoantibody Involvement in MS

While the cellular effects of B cells have only shifted into focus recently, it has been assumed for a long time that autoantibodies contribute to the pathogenesis of MS. *In vivo*, antibody and complement deposition has been shown to be present in a subset of MS lesions [27] both in chronic disease and early active lesions [48]. In addition, it has been shown that it is only those patients with evidence of antibody deposition in lesions that respond to plasmapheresis [49]. *In vitro*, studying the effect of serum antibodies from MS patients in myelinating cultures revealed induction of demyelination and, more rarely, axonal injury [50].

### 3.2. Antigen-Screening Approaches

A variety of antigen-screening approaches with antibodies from serum and CSF have been conducted, including immunohistochemical staining of brain sections, western blotting of one- or two-dimensionally separated proteins, immunoprecipitation of brain tissue or cell lysates, peptide and protein microarrays, and phage display and immunofluorescent labeling of transfected cells by flow cytometry [51,52]. Various aspects complicate the quest for the autoantigen target(s): first, it has been shown that not only the native conformation of the protein is crucial for the detection of disease-specific antibodies, but also that the various posttranslational modifications (glycosylation, phosphorylation, citrullination, and others) complicate the autoantigen identification; second, the pathogenic autoantibodies constitute only a minor part of the autoantibody repertoire, which has been experimentally approached by enriching for potential pathogenic autoantibodies from MS lesions or generating monoclonal antibodies from CNS-resident cells [16,53,54]. However, despite the vast effort that has been expended over the last decades in the field, the search for the antigen(s) in multiple sclerosis is still open.

### 3.3. Potential Autoantigen Targets

#### 3.3.1. Myelin Oligodendrocyte Glycoprotein (MOG) and Aquaporin-4 (AQP4)

The best-studied autoantigen in the field is MOG. MOG is a myelin glycoprotein that is located at the uttermost lamellae of the myelin sheath, making it easily accessible to potential autoantibodies. While the pathogenic role of anti-MOG antibodies in EAE is undisputed, the role of anti-MOG antibodies in MS patients has been controversially discussed over decades [55,56]. This has been fueled by the use of various different assays using recombinant, non-conformational MOG. More recently, with the use of different cell-based assays, anti-MOG antibodies could be identified in a subgroup of pediatric ADEM (acute disseminated encephalomyelitis), CIS (clinically isolated syndrome), or MS [57,58,59,60,61,62,63,64,65,66,67,68,69] and were shown to correlate with the disease course [63,70]. Moreover, it was shown that these antibodies are of the complement-activating IgG1 isotype [58,63], were able to exhibit complement-dependent cytotoxicity on MOG-expressing cells [71], and have a functional effect on the oligodendrocyte cytoskeleton [72], suggesting that these antibodies are potentially pathogenic. Analysis of the recognized epitopes revealed that the antibodies are directed against distinct epitopes without intramolecular epitope-spreading during disease course [73]. The fact that the presence of anti-MOG antibodies was negatively related to age [63] tempts one to speculate that the age of disease onset, *i.e.*, an ongoing myelination process in children, influences the autoantigen target.

Even more recently, anti-MOG antibodies have also been identified in a subgroup of AQP4-seronegative pediatric and adult patients with neuromyelitis optica spectrum disorder (NMOSD) presenting with a distinct and more benign clinical phenotype with strong correlation to uni-/bilateral recurrent optic neuritis [68,71,72,74,75,76,77,78]. Transfer of these antibodies to the mouse brain revealed reversible lesions fitting into the clinical picture of a reversible clinical demyelinating syndrome [79]. Future studies are needed to further study the pathogenic relevance of the antibodies and to address the question of whether these MOG-seropositive patients represent a unique disease entity [80].

While the role of anti-MOG antibodies in the pathogenesis of demyelination is still to be determined, the presence and diagnostic significance of AQP4 antibodies in NMOSD is undisputed [81,82,83,84,85]. In animal models, it has been shown that AQP4 antibodies can bind to astrocytes and lead to characteristic pathological features reminiscent of NMO (AQP4 and astrocyte loss, granulocytic infiltrates, T cells and activated macrophages/microglia cells, extensive immunoglobulin and complement deposition on astrocyte processes of the perivascular and superficial glia limitans) [84,86]. In fact, the discovery of AQP4 as a biomarker has marked a breakthrough in the understanding of the pathogenesis of the disease, being identified as the first confirmed autoantigen in a CNS demyelinating disease.

#### 3.3.2. Neurofascin and Contactin-2

The node of Ranvier has more recently received attention as the “Achilles heel” of the CNS [87]. Evidence for the perturbation of the nodal region was motivated by biomarker and pathological studies [88,89,90]. Two potential autoantigen targets, neurofascin and contactin-2, have been identified by a two-dimensional western blot approach with myelin glycoproteins derived from post-mortem human brain samples [91,92].

Neurofascin (NF) is a glycoprotein expressed both in the central and peripheral nervous systems. It exists in two isoforms: neurofascin 155 (NF155) is a myelin protein localized at the paranodal axo-glial junction, whereas NF186 is a neuronal protein exposed on the surface of myelinated axons at the axonal initial segment and node of Ranvier [93]. The neuronal isoform of neurofascin 186 exposed at the node is crucial for sodium channel clustering, while the glial isoform NF155 at the paranode is necessary for proper paranodal junction formation [94]. Autoantibodies against NF were detected in a subgroup of multiple sclerosis patients and, more recently, also in patients with combined central and peripheral demyelination [95]. *In vivo*, recombinant antibodies to NF were shown to induce axonal injury paralleled by reversible clinical deterioration in an experimental autoimmune encephalomyelitis model [91].

Contactin-2 is expressed both by glial cells and axons at the juxtaparanode. Contactin-2-specific T cells induced inflammatory cuffs in the gray matter of the spinal cord and the cortex in an animal model [92].

Both antigens provide a possible mechanism for axonal and gray matter injury which have been recognized only recently to be contributing to pathology early on during disease in parallel to demyelination [96,97].

#### 3.3.3. Inward-Rectifying Potassium Channel (KIR4.1)

Recently, antibodies against the inward rectifying potassium channel KIR4.1 were reported to be present in 47% of adult patients with MS and CIS and in an even higher proportion of pediatric patients, but in no healthy controls, using an enzyme-linked immunosorbent assay (ELISA) with either full-length KIR4.1 protein or peptide (amino acids 83–120) [98,99]. Subsequent independent studies using peptide ELISAs or a cell-based approach have failed to replicate these findings [100,101,102]. Future studies using the identical method as originally described need to be conducted to validate or refute the findings [103,104].

#### 3.3.4. Glycolipid-, Sulfatide-Specific, and Other Antibody Targets

In addition to the above-mentioned target antigens, numerous other antigen candidates have been described in MS patients including viral, microbial, and self-antigens [51,105,106]. More recently, there has been increasing evidence for (glycol-)lipid and sulfatide-specific antibodies [107,108].

## 4. B Cell-Directed Therapies—Towards Personalized Treatment

While substantial evidence for B cell involvement in MS comes from animal studies, the success of B cell-directed therapies illustrates a triumphant example of how translational medicine promotes basic findings into clinical applications, allowing for more diverse and personalized treatment options for MS patients. A variety of currently available treatments such as natalizumab, fingolimod, and alemtuzumab, as well as several anti-CD20 and anti-CD19 therapies currently being tested in clinical trials (anti-CD20: rituximab, ocrelizumab, ofatumumab; anti-CD19: MEDI-551; anti-BAFF-R: VAY736), have a substantial targeting effect on B cells (Table 1).

Although natalizumab exhibits its primary effect on the reduction of T cells in the CNS by blocking their egress into the CNS via alpha 4-integrin blockade, it has also been shown to reduce B cell counts in the CNS of MS patients [109]. In contrast, the effect of fingolimod is most pronounced in the peripheral blood, leading to a marked decrease of circulating B cells by sequestration in the secondary lymphoid organs, and it also directly alters proportions of B cell subpopulations in treated patients with MS [110]. Alemtuzumab, which has been recently approved for the treatment of active multiple sclerosis, is a monoclonal anti-CD52 antibody with a direct depleting effect on both B and T cells [111,112].

**Table 1:** Biological drugs targeting B cells or B cell-activating factors.

Biologic	Species & Isotype	Target
Rituximab	chimeric (murine/human) monoclonal IgG1	CD20
Ocrelizumab	humanized monoclonal IgG1	CD20
Ofatumumab	human monoclonal IgG1	CD20
MEDI-551	humanized monoclonal IgG1	CD19
VAY736	human monoclonal IgG1	BAFF-R
Atacicept	recombinant fusion protein TACI-Fc	BAFF/APRIL
Alemtuzumab	humanized monoclonal IgG1	CD52

Rituximab, ocrelizumab, and ofatumumab are different anti-CD20 depleting agents, which are currently in phase II and III trials with promising effects [113,114,115,116]. Two monoclonal antibodies targeting CD19 or B cell-activating factor-receptor (BAFF-R) on B cells, MEDI-551 [117] (ClinicalTrials.gov identifier: NCT01585766) and VAY736 (ClinicalTrials.gov identifier: NCT02038049), are currently enrolling patients in phase II trials.

A different approach with the soluble receptor atacicept (TACI-Fc) was based on inhibiting the B cell survival factors BAFF and APRIL (a proliferation-inducing ligand) (TNFSF13), which promote their function by binding to the transmembrane receptor TACI on B cells. Unexpectedly, treatment of MS patients led to disease exacerbation in the atacicept-treated groups [118] and had to be stopped. However, the mechanisms by which atacicept leads to disease exacerbation up to now remain elusive. In contrast to the other mentioned B cell-targeted therapies, atacicept additionally targets plasma cells, which are spared by CD19- and CD20-depleting antibodies, suggesting that protective regulatory plasma cells might have been the reason for the worsening of disease activity. Moreover, expression of BAFF receptor on neurons was described, allowing the speculation that atacicept also limits neuronal survival by decreasing BAFF levels [119]. Also, BAFF was shown to lead to the induction of IL-10-producing regulatory B cells [120], which might have been impaired by atacicept. Very recent studies investigating the function of TACI found that endogenous soluble TACI (sTACI) existed *in vivo*, which shares the decoy function of atacicept, suggesting that atacicept could interfere in the equilibrium between sTACI and BAFF involved in fine-tuning the balance between effector B cells and regulatory B cells [121].

## 5. Conclusions

During the last years, the understanding of the role of B cells in MS has progressed tremendously. Having been initially considered as antibody-producing cells with a contributing function in the pathogenesis, they are now recognized as main players in conjunction and close interplay with T cells, exhibiting stimulatory, regulatory, and pro- and anti-inflammatory capacities. Further characterization of diverse B cell phenotypes and their different functions in the initiation, propagation, and maintenance of inflammation is needed and will potentially pave the way for new treatment strategies. The identification of disease-specific autoantibodies against AQP4 and MOG in a subgroup of patients has not only advanced our pathogenic understanding, but it provides a valuable tool for clinical stratification. The search for other autoantigen targets is in progress, paving the way for the development of more specific, customized immunomodulatory/-suppressive treatments.

## Abbreviations

ADEM: acute disseminated encephalomyelitis
APC: antigen-presenting cell
APRIL: a proliferation-inducing ligand
AQP4: aquaporin-4
BAFF: B cell activating factor
CCL19: chemokine (C-C motif) ligand 19
CIS: clinically isolated syndrome
CNS: central nervous system
CSF: cerebrospinal fluid
EAE: experimental autoimmune encephalomyelitis
EBV: Epstein-Barr Virus
ELISA: enzyme-linked immunosorbent assay
Ig: immunoglobulin
IL: interleukin
MHC: major histocompatibility complex
MOG: myelin oligodendrocyte glycoprotein
MS: multiple sclerosis
NMO: neuromyelitis optica
NMOSD: NMO spectrum disease
OCBs: oligoclonal bands
TNF: tumor necrosis factor

## References

[1] Compston A., Coles A. (2008). Multiple sclerosis. Lancet.

[2] Hauser S.L., Oksenberg J.R. (2006). The neurobiology of multiple sclerosis: Genes, inflammation, and neurodegeneration. Neuron.

[3] Ascherio A., Munger K.L. (2007). Environmental risk factors for multiple sclerosis. Part I: The role of infection. Ann. Neurol..

[4] Ascherio A., Munger K.L. (2007). Environmental risk factors for multiple sclerosis. Part II: Noninfectious factors. Ann. Neurol..

[5] International Multiple Sclerosis Genetics Consortium (2007). Risk alleles for multiple sclerosis identified by a genomewide study. N. Engl. J. Med..

[6] Patsopoulos N.A., de Bakker P.I.W., Bayer Pharma MS Genetics Working Group, Steering Committees of Studies Evaluating IFNβ-1b and a CCR1-Antagonist, ANZgene Consortium, GeneMSA, International Multiple Sclerosis Genetics Consortium (2011). Genome-wide meta-analysis identifies novel multiple sclerosis susceptibility loci. Ann. Neurol..

[7] Lünemann J.D., Jelcić I., Roberts S., Lutterotti A., Tackenberg B., Martin R., Münz C. (2008). EBNA1-specific T cells from patients with multiple sclerosis cross react with myelin antigens and co-produce IFN-gamma and IL-2. J. Exp. Med..

[8] Sospedra M., Zhao Y., zur Hausen H., Muraro P.A., Hamashin C., de Villiers E.M., Pinilla C., Martin R. (2005). Recognition of conserved amino acid motifs of common viruses and its role in autoimmunity. PLoS Pathog..

[9] Krumbholz M., Derfuss T., Hohlfeld R., Meinl E. (2012). B cells and antibodies in multiple sclerosis pathogenesis and therapy. Nat. Rev. Neurol..

[10] Mayer M.C., Hohlfeld R., Meinl E. (2012). Viability of autoantibody-targets: How to tackle pathogenetic heterogeneity as an obstacle for treatment of multiple sclerosis. J. Neurol. Sci..

[11] Meinl E., Krumbholz M., Hohlfeld R. (2006). B lineage cells in the inflammatory central nervous system environment: Migration, maintenance, local antibody production, and therapeutic modulation. Ann. Neurol..

[12] Abraira V., Alvarez-Cermeño J.C., Arroyo R., Cámara C., Casanova B., Cubillo S., de Andrés C., Espejo C., Fernández O., Ferrer J. (2011). Utility of oligoclonal IgG band detection for MS diagnosis in daily clinical practice. J. Immunol. Methods.

[13] Thompson E.J., Kaufmann P., Rudge P. (1983). Sequential changes in oligoclonal patterns during the course of multiple sclerosis. J. Neurol. Neurosurg. Psychiatry.

[14] Walsh M.J., Tourtellotte W.W. (1986). Temporal invariance and clonal uniformity of brain and cerebrospinal IgG, IgA, and IgM in multiple sclerosis. J. Exp. Med..

[15] Mancuso R., Franciotta D., Rovaris M., Caputo D., Sala A., Hernis A., Agostini S., Calvo M., Clerici M. (2014). Effects of natalizumab on oligoclonal bands in the cerebrospinal fluid of multiple sclerosis patients: A longitudinal study. Mult. Scler..

[16] Owens G.P., Bennett J.L., Lassmann H., O'Connor K.C., Ritchie A.M., Shearer A., Lam C., Yu X., Birlea M., DuPree C. (2009). Antibodies produced by clonally expanded plasma cells in multiple sclerosis cerebrospinal fluid. Ann. Neurol..

[17] Obermeier B., Mentele R., Malotka J., Kellermann J., Kümpfel T., Wekerle H., Lottspeich F., Hohlfeld R., Dornmair K. (2008). Matching of oligoclonal immunoglobulin transcriptomes and proteomes of cerebrospinal fluid in multiple sclerosis. Nat. Med..

[18] Obermeier B., Lovato L., Mentele R., Brück W., Forne I., Imhof A., Lottspeich F., Turk K.W., Willis S.N., Wekerle H. (2011). Related B cell clones that populate the CSF and CNS of patients with multiple sclerosis produce CSF immunoglobulin. J. Neuroimmunol..

[19] Beltrán E., Obermeier B., Moser M., Coret F., Simó-Castelló M., Boscá I., Pérez-Miralles F., Villar L.M., Senel M., Tumani H. (2014). Intrathecal somatic hypermutation of IgM in multiple sclerosis and neuroinflammation. Brain.

[20] Qin Y., Duquette P., Zhang Y., Talbot P., Poole R., Antel J. (1998). Clonal expansion and somatic hypermutation of V(H) genes of B cells from cerebrospinal fluid in multiple sclerosis. J. Clin. Investig..

[21] Owens G.P., Ritchie A.M., Burgoon M.P., Williamson R.A., Corboy J.R., Gilden D.H. (2003). Single-cell repertoire analysis demonstrates that clonal expansion is a prominent feature of the B cell response in multiple sclerosis cerebrospinal fluid. J. Immunol..

[22] Bankoti J., Apeltsin L., Hauser S.L., Allen S., Albertolle M.E., Witkowska H.E., von Büdingen H.C. (2014). In multiple sclerosis, oligoclonal bands connect to peripheral B-cell responses. Ann. Neurol..

[23] Von Büdingen H.C., Kuo T.C., Sirota M., van Belle C.J., Apeltsin L., Glanville J., Cree B.A., Gourraud P.A., Schwartzburg A., Huerta G. (2012). B cell exchange across the blood-brain barrier in multiple sclerosis. J. Clin. Investig..

[24] Palanichamy A., Apeltsin L., Kuo T.C., Sirota M., Wang S., Pitts S.J., Sundar P.D., Telman D., Zhao L.Z., Derstine M. (2014). Immunoglobulin class-switched B cells form an active immune axis between CNS and periphery in multiple sclerosis. Sci. Transl. Med..

[25] Stern J.N.H., Yaari G., Vander Heiden J.A., Church G., Donahue W.F., Hintzen R.Q., Huttner A.J., Laman J.D., Nagra R.M., Nylander A. (2014). B cells populating the multiple sclerosis brain mature in the draining cervical lymph nodes. Sci. Transl. Med..

[26] Storch M.K., Piddlesden S., Haltia M., Iivanainen M., Morgan P., Lassmann H. (1998). Multiple sclerosis: *In situ* evidence for antibody- and complement-mediated demyelination. Ann. Neurol..

[27] Lucchinetti C., Brück W., Parisi J., Scheithauer B., Rodriguez M., Lassmann H. (2000). Heterogeneity of multiple sclerosis lesions: Implications for the pathogenesis of demyelination. Ann. Neurol..

[28] Serafini B., Rosicarelli B., Magliozzi R., Stigliano E., Aloisi F. (2004). Detection of ectopic B-cell follicles with germinal centers in the meninges of patients with secondary progressive multiple sclerosis. Brain Pathol..

[29] Magliozzi R., Howell O., Vora A., Serafini B., Nicholas R., Puopolo M., Reynolds R., Aloisi F. (2007). Meningeal B-cell follicles in secondary progressive multiple sclerosis associate with early onset of disease and severe cortical pathology. Brain.

[30] Howell O.W., Reeves C.A., Nicholas R., Carassiti D., Radotra B., Gentleman S.M., Serafini B., Aloisi F., Roncaroli F., Magliozzi R. (2011). Meningeal inflammation is widespread and linked to cortical pathology in multiple sclerosis. Brain.

[31] Haugen M., Frederiksen J.L., Degn M. (2014). B cell follicle-like structures in multiple sclerosis-with focus on the role of B cell activating factor. J. Neuroimmunol..

[32] Lovato L., Willis S.N., Rodig S.J., Caron T., Almendinger S.E., Howell O.W., Reynolds R., O'Connor K.C., Hafler D.A. (2011). Related B cell clones populate the meninges and parenchyma of patients with multiple sclerosis. Brain.

[33] Louveau A., Smirnov I., Keyes T.J., Eccles J.D., Rouhani S.J., Peske J.D., Derecki N.C., Castle D., Mandell J.W., Lee K.S. (2015). Structural and functional features of central nervous system lymphatic vessels. Nature.

[34] Hatterer E., Davoust N., Didier-Bazes M., Vuaillat C., Malcus C., Belin M.F., Nataf S. (2006). How to drain without lymphatics? Dendritic cells migrate from the cerebrospinal fluid to the B-cell follicles of cervical lymph nodes. Blood.

[35] Krumbholz M., Theil D., Derfuss T., Rosenwald A., Schrader F., Monoranu C.M., Kalled S.L., Hess D.M., Serafini B., Aloisi F. (2005). BAFF is produced by astrocytes and up-regulated in multiple sclerosis lesions and primary central nervous system lymphoma. J. Exp. Med..

[36] Krumbholz M., Theil D., Cepok S., Hemmer B., Kivisäkk P., Ransohoff R.M., Hofbauer M., Farina C., Derfuss T., Hartle C. (2006). Chemokines in multiple sclerosis: CXCL12 and CXCL13 up-regulation is differentially linked to CNS immune cell recruitment. Brain.

[37] Krumbholz M., Theil D., Steinmeyer F., Cepok S., Hemmer B., Hofbauer M., Farina C., Derfuss T., Junker A., Arzberger T. (2007). CCL19 is constitutively expressed in the CNS, up-regulated in neuroinflammation, active and also inactive multiple sclerosis lesions. J. Neuroimmunol..

[38] Magliozzi R., Columba-Cabezas S., Serafini B., Aloisi F. (2004). Intracerebral expression of CXCL13 and BAFF is accompanied by formation of lymphoid follicle-like structures in the meninges of mice with relapsing experimental autoimmune encephalomyelitis. J. Neuroimmunol..

[39] Khademi M., Kockum I., Andersson M.L., Iacobaeus E., Brundin L., Sellebjerg F., Hillert J., Piehl F., Olsson T. (2011). Cerebrospinal fluid CXCL13 in multiple sclerosis: A suggestive prognostic marker for the disease course. Mult. Scler..

[40] Molnarfi N., Schulze-Topphoff U., Weber M.S., Patarroyo J.C., Prod’homme T., Varrin-Doyer M., Shetty A., Linington C., Slavin A.J., Hidalgo J. (2013). MHC class II-dependent B cell APC function is required for induction of CNS autoimmunity independent of myelin-specific antibodies. J. Exp. Med..

[41] Pierson E.R., Stromnes I.M., Goverman J.M. (2014). B cells promote induction of experimental autoimmune encephalomyelitis by facilitating reactivation of T cells in the central nervous system. J. Immunol..

[42] Barr T.A., Shen P., Brown S., Lampropoulou V., Roch T., Lawrie S., Fan B., O'Connor R.A., Anderton S.M., Bar-Or A. (2012). B cell depletion therapy ameliorates autoimmune disease through ablation of IL-6-producing B cells. J. Exp. Med..

[43] Shen P., Fillatreau S. (2015). Antibody-independent functions of B cells: A focus on cytokines. Nat. Rev. Immunol..

[44] Fillatreau S., Sweenie C.H., McGeachy M.J., Gray D., Anderton S.M. (2002). B cells regulate autoimmunity by provision of IL-10. Nat. Immunol..

[45] Shen P., Roch T., Lampropoulou V., O'Connor R.A., Stervbo U., Hilgenberg E., Ries S., Dang V. D., Jaimes Y., Daridon C. (2014). IL-35-producing B cells are critical regulators of immunity during autoimmune and infectious diseases. Nature.

[46] Ries S., Hilgenberg E., Lampropoulou V., Shen P., Dang V.D., Wilantri S., Sakwa I., Fillatreau S. (2014). B-type suppression: A role played by “regulatory B cells” or “regulatory plasma cells”?. Eur. J. Immunol..

[47] Yoshizaki A., Miyagaki T., DiLillo D.J., Matsushita T., Horikawa M., Kountikov E.I., Spolski R., Poe J.C., Leonard W.J., Tedder T.F. (2012). Regulatory B cells control T-cell autoimmunity through IL-21-dependent cognate interactions. Nature.

[48] Metz I., Weigand S.D., Popescu B.F.G., Frischer J.M., Parisi J.E., Guo Y., Lassmann H., Brück W., Lucchinetti C.F. (2014). Pathologic heterogeneity persists in early active multiple sclerosis lesions. Ann. Neurol..

[49] Keegan M., König F., McClelland R., Brück W., Morales Y., Bitsch A., Panitch H., Lassmann H., Weinshenker B., Rodriguez M. (2005). Relation between humoral pathological changes in multiple sclerosis and response to therapeutic plasma exchange. Lancet.

[50] Elliott C., Lindner M., Arthur A., Brennan K., Jarius S., Hussey J., Chan A., Stroet A., Olsson T., Willison H. (2012). Functional identification of pathogenic autoantibody responses in patients with multiple sclerosis. Brain.

[51] Schirmer L., Srivastava R., Hemmer B. (2014). To look for a needle in a haystack: The search for autoantibodies in multiple sclerosis. Mult. Scler..

[52] Derfuss T., Meinl E. (2012). Identifying autoantigens in demyelinating diseases: Valuable clues to diagnosis and treatment?. Curr. Opin. Neurol..

[53] Von Büdingen H.C., Harrer M.D., Kuenzle S., Meier M., Goebels N. (2008). Clonally expanded plasma cells in the cerebrospinal fluid of MS patients produce myelin-specific antibodies. Eur. J. Immunol..

[54] O'Connor K.C., Appel H., Bregoli L., Call M.E., Catz I., Chan J.A., Moore N.H., Warren K.G., Wong S.J., Hafler D.A. (2005). Antibodies from inflamed central nervous system tissue recognize myelin oligodendrocyte glycoprotein. J. Immunol..

[55] Berger T., Rubner P., Schautzer F., Egg R., Ulmer H., Mayringer I., Dilitz E., Deisenhammer F., Reindl M. (2003). Antimyelin antibodies as a predictor of clinically definite multiple sclerosis after a first demyelinating event. N. Engl. J. Med..

[56] Kuhle J., Pohl C., Mehling M., Edan G., Freedman M.S., Hartung H.P., Polman C.H., Miller D.H., Montalban X., Barkhof F. (2007). Lack of association between antimyelin antibodies and progression to multiple sclerosis. N. Engl. J. Med..

[57] O'Connor K.C., McLaughlin K.A., de Jager P.L., Chitnis T., Bettelli E., Xu C., Robinson W.H., Cherry S.V., Bar-Or A., Banwell B. (2007). Self-antigen tetramers discriminate between myelin autoantibodies to native or denatured protein. Nat. Med..

[58] McLaughlin K.A., Chitnis T., Newcombe J., Franz B., Kennedy J., McArdel S., Kuhle J., Kappos L., Rostásy K., Pohl D. (2009). Age-dependent B cell autoimmunity to a myelin surface antigen in pediatric multiple sclerosis. J. Immunol..

[59] Brilot F., Dale R.C., Selter R.C., Grummel V., Kalluri S.R., Aslam M., Busch V., Zhou D., Cepok S., Hemmer B. (2009). Antibodies to native myelin oligodendrocyte glycoprotein in children with inflammatory demyelinating central nervous system disease. Ann. Neurol..

[60] Rostásy K., Mader S., Schanda K., Huppke P., Gärtner J., Kraus V., Karenfort M., Tibussek D., Blaschek A., Bajer-Kornek B. (2012). Anti-myelin oligodendrocyte glycoprotein antibodies in pediatric patients with optic neuritis. Arch. Neurol..

[61] Baumann M., Sahin K., Lechner C., Hennes E.M., Schanda K., Mader S., Karenfort M., Selch C., Häusler M., Eisenkölbl A. (2015). Clinical and neuroradiological differences of paediatric acute disseminating encephalomyelitis with and without antibodies to the myelin oligodendrocyte glycoprotein. J. Neurol. Neurosurg. Psychiatry.

[62] Hacohen Y., Absoud M., Woodhall M., Cummins C., de Goede C.G., Hemingway C., Jardine P.E., Kneen R., Pike M.G., Whitehouse W.P. (2014). Autoantibody biomarkers in childhood-acquired demyelinating syndromes: Results from a national surveillance cohort. J. Neurol. Neurosurg. Psychiatry.

[63] Pröbstel A.K., Dornmair K., Bittner R., Sperl P., Jenne D., Magalhaes S., Villalobos A., Breithaupt C., Weissert R., Jacob U. (2011). Antibodies to MOG are transient in childhood acute disseminated encephalomyelitis. Neurology.

[64] Dale R.C., Tantsis E.M., Merheb V., Kumaran R.Y.A., Sinmaz N., Pathmanandavel K., Ramanathan S., Booth D.R., Wienholt L.A., Prelog K. (2014). Antibodies to MOG have a demyelination phenotype and affect oligodendrocyte cytoskeleton. Neurol. Neuroimmunol. Neuroinflamm..

[65] Huppke P., Rostásy K., Karenfort M., Huppke B., Seidl R., Leiz S., Reindl M., Gärtner J. (2013). Acute disseminated encephalomyelitis followed by recurrent or monophasic optic neuritis in pediatric patients. Mult. Scler..

[66] Selter R.C., Brilot F., Grummel V., Kraus V., Cepok S., Dale R.C., Hemmer B. (2010). Antibody responses to EBV and native MOG in pediatric inflammatory demyelinating CNS diseases. Neurology.

[67] Ketelslegers I.A., van Pelt D.E., Bryde S. (2015). Anti-MOG antibodies plead against MS diagnosis in an Acquired Demyelinating Syndromes cohort. Mult. Scler..

[68] Rostasy K., Mader S., Hennes E.M., Schanda K., Gredler V., Guenther A., Blaschek A., Korenke C., Pritsch M., Pohl D. (2013). Persisting myelin oligodendrocyte glycoprotein antibodies in aquaporin-4 antibody negative pediatric neuromyelitis optica. Mult. Scler..

[69] Hacohen Y., Absoud M., Deiva K., Hemingway C., Nytrova P., Woodhall M., Palace J., Wassmer E., Tardieu M., Vincent A. (2015). Myelin oligodendrocyte glycoprotein antibodies are associated with a non-MS course in children. Neurol. Neuroimmunol. Neuroinflamm..

[70] Di Pauli F., Mader S., Rostásy K., Schanda K., Bajer-Kornek B., Ehling R., Deisenhammer F., Reindl M., Berger T. (2011). Temporal dynamics of anti-MOG antibodies in CNS demyelinating diseases. Clin. Immunol..

[71] Mader S., Gredler V., Schanda K., Rostásy K., Dujmovic I., Pfaller K., Lutterotti A., Jarius S., di Pauli F., Kuenz B. (2011). Complement activating antibodies to myelin oligodendrocyte glycoprotein in neuromyelitis optica and related disorders. J. Neuroinflam..

[72] Ramanathan S., Reddel S.W., Henderson A., Parratt J.D.E., Barnett M., Gatt P.N., Merheb V., Kumaran R.Y.A., Pathmanandavel K., Sinmaz N. (2014). Antibodies to myelin oligodendrocyte glycoprotein in bilateral and recurrent optic neuritis. Neurol. Neuroimmunol. Neuroinflamm..

[73] Mayer M.C., Breithaupt C., Reindl M., Schanda K., Rostásy K., Berger T., Dale R.C., Brilot F., Olsson T., Jenne D. (2013). Distinction and temporal stability of conformational epitopes on myelin oligodendrocyte glycoprotein recognized by patients with different inflammatory central nervous system diseases. J. Immunol..

[74] Kitley J., Woodhall M., Waters P., Leite M.I., Devenney E., Craig J., Palace J., Vincent A. (2012). Myelin-oligodendrocyte glycoprotein antibodies in adults with a neuromyelitis optica phenotype. Neurology.

[75] Kitley J., Waters P., Woodhall M., Leite M.I., Murchison A., George J., Küker W., Chandratre S., Vincent A., Palace J. (2014). Neuromyelitis optica spectrum disorders with aquaporin-4 and myelin-oligodendrocyte glycoprotein antibodies: A comparative study. JAMA Neurol..

[76] Sato D.K., Callegaro D., Lana-Peixoto M.A., Waters P.J., Jorge F.M., de H., Takahashi T., Nakashima I., Apostolos-Pereira S.L., Talim N. (2014). Distinction between MOG antibody-positive and AQP4 antibody-positive NMO spectrum disorders. Neurology.

[77] Höftberger R., Sepulveda M., Armangue T., Blanco Y., Rostásy K., Cobo Calvo A., Olascoaga J., Ramió-Torrentà L., Reindl M., Benito-León J. (2015). Antibodies to MOG and AQP4 in adults with neuromyelitis optica and suspected limited forms of the disease. Mult. Scler..

[78] Pröbstel A.-K., Rudolf G., Dornmair K., Collongues N., Chanson J.-B., Sanderson N.S., Lindberg R.L., Kappos L., de Sèze J., Derfuss T. (2015). Anti-MOG antibodies are present in a subgroup of patients with a neuromyelitis optica phenotype. J. Neuroinflamm..

[79] Saadoun S., Waters P., Owens G.P., Bennett J.L., Vincent A., Papadopoulos M.C. (2014). Neuromyelitis optica MOG-IgG causes reversible lesions in mouse brain. Acta Neuropathol. Commun..

[80] Zamvil S.S., Slavin A.J. (2015). Does MOG Ig-positive AQP4-seronegative opticospinal inflammatory disease justify a diagnosis of NMO spectrum disorder?. Neurol. Neuroimmunol. Neuroinflamm..

[81] Lennon V.A., Wingerchuk D.M., Kryzer T.J., Pittock S.J., Lucchinetti C.F., Fujihara K., Nakashima I., Weinshenker B.G. (2004). A serum autoantibody marker of neuromyelitis optica: Distinction from multiple sclerosis. Lancet.

[82] Wingerchuk D.M., Lennon V.A., Pittock S.J., Lucchinetti C.F., Weinshenker B.G. (2006). Revised diagnostic criteria for neuromyelitis optica. Neurology.

[83] Wingerchuk D.M., Lennon V.A., Lucchinetti C.F., Pittock S.J., Weinshenker B.G. (2007). The spectrum of neuromyelitis optica. Lancet Neurol.

[84] Bradl M., Misu T., Takahashi T., Watanabe M., Mader S., Reindl M., Adzemovic M., Bauer J., Berger T., Fujihara K. (2009). Neuromyelitis optica: Pathogenicity of patient immunoglobulin *in vivo*. Ann. Neurol..

[85] De Seze J., Stojkovic T., Ferriby D., Gauvrit J.Y., Montagne C., Mounier-Vehier F., Verier A., Pruvo J.P., Hache J.C., Vermersch P. (2002). Devic’s neuromyelitis optica: Clinical, laboratory, MRI and outcome profile. J. Neurol. Sci..

[86] Bennett J.L., Lam C., Kalluri S.R., Saikali P., Bautista K., DuPree C., Glogowska M., Case D., Antel J.P., Owens G.P. (2009). Intrathecal pathogenic anti-aquaporin-4 antibodies in early neuromyelitis optica. Ann. Neurol..

[87] Derfuss T., Linington C., Hohlfeld R., Meinl E. (2010). Axo-glial antigens as targets in multiple sclerosis: Implications for axonal and grey matter injury. J. Mol. Med..

[88] Dhaunchak A.S., Becker C., Schulman H., de Faria O., Rajasekharan S., Banwell B., Colman D.R., Bar-Or A. (2012). Canadian Pediatric Demyelinating Disease Group Implication of perturbed axoglial apparatus in early pediatric multiple sclerosis. Ann. Neurol..

[89] Howell O.W., Palser A., Polito A., Melrose S., Zonta B., Scheiermann C., Vora A.J., Brophy P.J., Reynolds R. (2006). Disruption of neurofascin localization reveals early changes preceding demyelination and remyelination in multiple sclerosis. Brain.

[90] Wolswijk G., Balesar R. (2003). Changes in the expression and localization of the paranodal protein Caspr on axons in chronic multiple sclerosis. Brain.

[91] Mathey E.K., Derfuss T., Storch M.K., Williams K.R., Hales K., Woolley D.R., Al-Hayani A., Davies S.N., Rasband M.N., Olsson T. (2007). Neurofascin as a novel target for autoantibody-mediated axonal injury. J. Exp. Med..

[92] Derfuss T., Parikh K., Velhin S., Braun M., Mathey E., Krumbholz M., Kümpfel T., Moldenhauer A., Rader C., Sonderegger P. (2009). Contactin-2/TAG-1-directed autoimmunity is identified in multiple sclerosis patients and mediates gray matter pathology in animals. Proc. Natl. Acad. Sci. USA.

[93] Sherman D.L., Tait S., Melrose S., Johnson R., Zonta B., Court F.A., Macklin W.B., Meek S., Smith A.J.H., Cottrell D.F. (2005). Neurofascins are required to establish axonal domains for saltatory conduction. Neuron.

[94] Feinberg K., Eshed-Eisenbach Y., Frechter S., Amor V., Salomon D., Sabanay H., Dupree J.L., Grumet M., Brophy P.J., Shrager P. (2010). A glial signal consisting of gliomedin and NrCAM clusters axonal Na^+^ channels during the formation of nodes of Ranvier. Neuron.

[95] Kawamura N., Yamasaki R., Yonekawa T., Matsushita T., Kusunoki S., Nagayama S., Fukuda Y., Ogata H., Matsuse D., Murai H. (2013). Anti-neurofascin antibody in patients with combined central and peripheral demyelination. Neurology.

[96] Seehusen F., Baumgärtner W. (2010). Axonal pathology and loss precede demyelination and accompany chronic lesions in a spontaneously occurring animal model of multiple sclerosis. Brain Pathol..

[97] Lucchinetti C.F., Popescu B.F.G., Bunyan R.F., Moll N.M., Roemer S.F., Lassmann H., Brück W., Parisi J.E., Scheithauer B.W., Giannini C. (2011). Inflammatory cortical demyelination in early multiple sclerosis. N. Engl. J. Med..

[98] Srivastava R., Aslam M., Kalluri S.R., Schirmer L., Buck D., Tackenberg B., Rothhammer V., Chan A., Gold R., Berthele A. (2012). Potassium channel KIR4.1 as an immune target in multiple sclerosis. N. Engl. J. Med..

[99] Kraus V., Srivastava R., Kalluri S.R., Seidel U., Schuelke M., Schimmel M., Rostásy K., Leiz S., Hosie S., Grummel V. (2014). Potassium channel KIR4.1-specific antibodies in children with acquired demyelinating CNS disease. Neurology.

[100] Nerrant E., Salsac C., Charif M., Ayrignac X., Carra-Dalliere C., Castelnovo G., Goulabchand R., Tisseyre J., Raoul C., Eliaou J.F. (2014). Lack of confirmation of anti-inward rectifying potassium channel 4.1 antibodies as reliable markers of multiple sclerosis. Mult. Scler..

[101] Brickshawana A., Hinson S.R., Romero M.F., Lucchinetti C.F., Guo Y., Buttmann M., Mckeon A., Pittock S.J., Chang M.H., Chen A.P. (2014). Investigation of the KIR4.1 potassium channel as a putative antigen in patients with multiple sclerosis: A comparative study. Lancet Neurol..

[102] Brill L., Goldberg L., Karni A., Petrou P., Abramsky O., Ovadia H., Ben-Hur T., Karussis D., Vaknin-Dembinsky A. (2015). Increased anti-KIR4.1 antibodies in multiple sclerosis: Could it be a marker of disease relapse?. Mult. Scler..

[103] Filippi M., Rocca M.A., Lassmann H. (2014). KIR4.1: Another misleading expectation in multiple sclerosis?. Lancet Neurol..

[104] Hemmer B. (2015). Antibodies to the inward rectifying potassium channel 4.1 in multiple sclerosis: Different methodologies-conflicting results?. Mult. Scler..

[105] Derfuss T., Gürkov R., Then Bergh F., Goebels N., Hartmann M., Barz C., Wilske B., Autenrieth I., Wick M., Hohlfeld R. (2001). Intrathecal antibody production against Chlamydia pneumoniae in multiple sclerosis is part of a polyspecific immune response. Brain.

[106] Derfuss T., Hohlfeld R., Meinl E. (2005). Intrathecal antibody (IgG) production against human herpesvirus type 6 occurs in about 20% of multiple sclerosis patients and might be linked to a polyspecific B-cell response. J. Neurol..

[107] Brennan K.M., Galban-Horcajo F., Rinaldi S., O'Leary C.P., Goodyear C.S., Kalna G., Arthur A., Elliot C., Barnett S., Linington C. (2011). Lipid arrays identify myelin-derived lipids and lipid complexes as prominent targets for oligoclonal band antibodies in multiple sclerosis. J. Neuroimmunol..

[108] Kanter J.L., Narayana S., Ho P.P., Catz I., Warren K.G., Sobel R.A., Steinman L., Robinson W.H. (2006). Lipid microarrays identify key mediators of autoimmune brain inflammation. Nat. Med..

[109] Stüve O., Marra C.M., Jerome K.R., Cook L., Cravens P.D., Cepok S., Frohman E.M., Phillips J.T., Arendt G., Hemmer B. (2006). Immune surveillance in multiple sclerosis patients treated with natalizumab. Ann. Neurol..

[110] Miyazaki Y., Niino M., Fukazawa T., Takahashi E., Nonaka T., Amino I., Tashiro J., Minami N., Fujiki N., Doi S. (2014). Suppressed pro-inflammatory properties of circulating B cells in patients with multiple sclerosis treated with fingolimod, based on altered proportions of B-cell subpopulations. Clin. Immunol..

[111] Coles A.J., Twyman C.L., Arnold D.L., Cohen J.A., Confavreux C., Fox E.J., Hartung H.P., Havrdova E., Selmaj K.W., Weiner H.L. (2012). CARE-MS II investigators Alemtuzumab for patients with relapsing multiple sclerosis after disease-modifying therapy: A randomised controlled phase 3 trial. Lancet.

[112] Cohen J.A., Coles A.J., Arnold D.L., Confavreux C., Fox E.J., Hartung H.P., Havrdova E., Selmaj K.W., Weiner H.L., Fisher E. (2012). CARE-MS I investigators Alemtuzumab *versus* interferon beta 1a as first-line treatment for patients with relapsing-remitting multiple sclerosis: A randomised controlled phase 3 trial. Lancet.

[113] Hauser S.L., Waubant E., Arnold D.L., Vollmer T., Antel J., Fox R.J., Bar-Or A., Panzara M., Sarkar N., Agarwal S. (2008). HERMES Trial Group B-cell depletion with rituximab in relapsing-remitting multiple sclerosis. N. Engl. J. Med..

[114] Hawker K., O'Connor P., Freedman M.S., Calabresi P.A., Antel J., Simon J., Hauser S., Waubant E., Vollmer T., Panitch H. (2009). OLYMPUS trial group Rituximab in patients with primary progressive multiple sclerosis: Results of a randomized double-blind placebo-controlled multicenter trial. Ann. Neurol..

[115] Kappos L., Li D., Calabresi P.A., O'Connor P., Bar-Or A., Barkhof F., Yin M., Leppert D., Glanzman R., Tinbergen J. (2011). Ocrelizumab in relapsing-remitting multiple sclerosis: A phase 2, randomised, placebo-controlled, multicentre trial. Lancet.

[116] Sorensen P.S., Lisby S., Grove R., Derosier F., Shackelford S., Havrdova E., Drulovic J., Filippi M. (2014). Safety and efficacy of ofatumumab in relapsing-remitting multiple sclerosis: A phase 2 study. Neurology.

[117] Herbst R., Wang Y., Gallagher S., Mittereder N., Kuta E., Damschroder M., Woods R., Rowe D.C., Cheng L., Cook K. (2010). B-cell depletion *in vitro* and *in vivo* with an afucosylated anti-CD19 antibody. J. Pharmacol. Exp. Ther..

[118] Kappos L., Hartung H.P., Freedman M.S., Boyko A., Radü E.W., Mikol D.D., Lamarine M., Hyvert Y., Freudensprung U., Plitz T. (2014). ATAMS Study Group Atacicept in multiple sclerosis (ATAMS): A randomised, placebo-controlled, double-blind, phase 2 trial. Lancet Neurol..

[119] Tada S., Yasui T., Nakatsuji Y., Okuno T., Koda T., Mochizuki H., Sakoda S., Kikutani H. (2013). BAFF controls neural cell survival through BAFF receptor. PLoS ONE.

[120] Yang M., Sun L., Wang S., Ko K.H., Xu H., Zheng B.J., Cao X., Lu L. (2010). Novel function of B cell-activating factor in the induction of IL-10-producing regulatory B cells. J. Immunol..

[121] Hoffmann F.S., Kuhn P.H., Laurent S.A., Hauck S.M., Berer K., Wendlinger S.A., Krumbholz M., Khademi M., Olsson T., Dreyling M. (2015). The immunoregulator soluble TACI is released by ADAM10 and reflects B cell activation in autoimmunity. J. Immunol..

